# Cardiovascular disease risk prevention services by pharmacists in Saudi Arabia: what do policymakers and opinion leaders think?

**DOI:** 10.1186/s40545-021-00319-6

**Published:** 2021-05-06

**Authors:** Hadi A. Almansour, Nouf M. Aloudah, Tariq M. Alhawassi, Betty Chaar, Ines Krass, Bandana Saini

**Affiliations:** 1 School of Pharmacy, Faculty of Medicine and Health, The University of Sydney, Building Number A15, Sydney, NSW 2006 Australia; 2 College of Pharmacy, King Saud University, Riyadh, Saudi Arabia; 3 Medication Safety Research Chair, College of Pharmacy, King Saud University, Riyadh, Saudi Arabia; 4 Woolcock Institute of Medical Research, Sydney, NSW Australia

**Keywords:** CVD risk prevention services, Pharmacists, Policymakers, System reform, Pharmacy services

## Abstract

**Background:**

Cardiovascular disease (CVD) is an emerging contributor to national morbidity and mortality in Saudi Arabia. CVD risk prevention services are limited, particularly with an over-utilised public health sector and an under-utilised and under-resourced primary care sector. Globally, there is evidence that community pharmacists can play a key role in CVD prevention within primary care. However, the perspectives of policymakers and opinion leaders are critical to successful translation of evidence into practice. Thus, the aim was to engage policymakers and professional leaders in discussions about implementing high-quality CVD risk prevention services in community pharmacy.

**Methods:**

Qualitative semi-structured interviews were conducted, audio-recorded and transcribed verbatim. All transcripts were thematically analysed.

**Results:**

A total of 23 participants (87% male) from government and non-government sectors were interviewed. Of these, almost 65% had pharmacy qualifications. Limited provision of CVD risks preventative services in primary care was acknowledged by most participants and building community pharmacists’ capacity to assist in preventive health services was viewed favourably as one way of improving the status quo. The data yielded four key themes: (1) future pharmacy CVD health service models; (2) demonstrable outcomes; (3) professional engagement and advocacy; and (4) implementability. CVD health services roles (health screening, primary and secondary prevention services), pragmatic factors and tiered models of care (minimal, medium, and comprehensive pharmacist involvement) were discussed. The need for humanistic, clinical, and cost effectiveness outcomes to be demonstrated and active involvement of professional bodies were deemed important for such services to be sustainable. Professional pharmacy governance to develop pharmacy careers and workforce, pharmacy curricular reform and ongoing education were posed as key success factors for novel pharmacy roles. Practice policies, standards, and guidelines were seen as required to adhere to stringent quality control for future pharmacy services provision. Participant’s implementation vision for such services included scalability, affordability, access, adoption and health system reform. Most discussions focused on the need for structural improvement with limited input regarding processes or outcomes required to establish such models.

**Conclusions:**

Most participants favoured pharmacy-based CVD risk prevention services, despite the variability in proposed service models. However, prior to developing such services, support structures at the health system and health professional level are needed as well as building public support and acceptability for pharmacy services.

**Supplementary Information:**

The online version contains supplementary material available at 10.1186/s40545-021-00319-6.

## Introduction

Human health and longevity gains acquired through scientific advances against infectious disease over the previous century are increasingly overshadowed by globally rising non-communicable disease burdens. This has created increasing demand for equitable, accessible and affordable healthcare which places immense pressure on governments worldwide [1–4], and indeed, this is the case in Saudi Arabia. Non-communicable diseases (NCDs) now contribute significantly to national disease burdens globally [5], and in Saudi Arabia, these account for approximately 73% of total mortality [6, 7]. Saudi Arabia has a nationalised healthcare system with government supported care provided through many public health channels with increasing participation of the private sector in recent years [8]. Recently, in Saudi Arabia, healthcare reforms within the national transformation program 2020 (part of Vision 2030, 2016) were proposed by the Ministry of Health (MoH) [9]. The aim of these reforms was to develop a Saudi model that meets community demands for high quality and efficient healthcare, and promote preventive action against non-communicable health risk factors [9]. Achieving the Vision 2030 goals in Saudi healthcare requires innovative exploration and research into healthcare models that would achieve these goals.

One method of achieving better returns on healthcare investment could be to make better use of the expertise of allied healthcare professionals (HCPs), such as pharmacists, nurses, and dietitians, especially when there is an upsurge of demand on primary care physicians (e.g., General Practitioners (GP)) [10]. This parsimonious approach is recommended by the WHO for several countries. Utilising allied HCPs and task-shifting some roles or services may, apart from economic advantage, ease the pressure on the healthcare system and optimise patient care and health outcomes [1, 11]. Pharmacists in community settings are highly accessible, well-trained professionals who, given the opportunity, can provide healthcare advice, point of care testing (POCT), screening, management/referral of ‘at risk’ patients besides ensuring appropriate use of medicines [5, 10]. In some countries, the utilisation of community pharmacists has been shown to strengthen the primary care setting, e.g., by task-shifting chronic disease risk prevention and management from tertiary to community settings [10]. Utilising community pharmacies also presents a particularly efficient healthcare model given that the retail nature of pharmacy requires private sector infrastructure, which can be effectively used to launch public health services without incurring public health infrastructure budgets. This would be especially important in the context of chronic diseases, which can be managed at a primary healthcare level and where population gains are realisable with preventive health and chronic disease management programs.

Amongst NCDs, cardiovascular diseases (CVD) are major causes of death in Saudi Arabia [7]. There is a high and rising prevalence of CVD and metabolic risk factors among the Saudi population [7], which requires critical investment into CVD risk prevention services at the community level. Research from various countries has demonstrated that pharmacists as primary HCPs can play a role in CVD prevention services [12–16]. This has led pharmacy professional bodies globally to make key recommendations to policymakers for the deeper integration of community pharmacy within primary care, and to develop legislative and remunerative pathways allowing sustainable multiple health services delivery in pharmacies [10].

In the Saudi context, the feasibility of developing pharmacy CVD risk prevention services has been explored through qualitative exploratory work, which gauged the perspective of stakeholders including health consumers (primary stakeholders), as well as community/hospital pharmacists and physicians (secondary stakeholders) [17–19]. Pharmacy-based CVD risk prevention services appeared acceptable to most stakeholder groups. Pharmacists appeared willing to consider the delivery of such services, but recommended that legislative and professional frameworks, public promotion of pharmacy roles, and ministerial approval for such models were essential prerequisites. Most pharmacist participants also expressed a willingness to collaborate with primary care physicians in the provision of such services. Consumers’ current health service expectations from pharmacies were low, but they appeared receptive to future services to help them prevent and manage CVD risk. Clearly overcoming barriers at several tiers of the health system would be needed to implement pharmacist-provided CVD prevention services [17–19]. The stakeholder mosaic explored above did not include policymakers or pharmacy opinion leaders (key stakeholders), which is a gap that needed to be addressed prior to moving forward with designing or implementing such services.

The policymakers’ stance around pharmacy services for CVD is particularly important as recently, and several policies allowing community pharmacists to provide services were published by the Saudi MoH. However, the details for wide scale and sustainable implementation, such as funding and ministerial support, were not clearly articulated in these publications [20]. The aim of this study, therefore, was to explore policymaker and industry leader perspectives and recommendations around the development of high-quality CVD risk prevention (screening and management) services in community pharmacy. Understanding the vision, support, and direction from these national level leaders and policymakers is required to drive consumer, health professional and health system changes necessary for the implementation of novel pharmacy-based services [21].

## Methods

The methods used in this qualitative study are reported following the Consolidated Criteria for Reporting Qualitative Studies (COREQ) checklist (Additional file 1: Appendix A) [22]. Ethical approval for the study protocol was obtained from the University of Sydney Human Research Ethics Committee (HREC) (2017/614) and King Saud University Institutional Review Board (IRB) (E-18–3470).

### Sampling and recruitment

Purposive sampling was used to target stakeholders with experience in health/pharmaceutical policy implementation in Saudi Arabia. Participants were included if they were 1) key policymakers/industry leaders or academics who may be representatives of organisations that are likely to be involved in future decisions about role diversification within the pharmacy health workforce, or 2) if they were involved in implementation of community-based programs to improve CVD risk prevention and management. Through the clinical, research and industry experience of our local Saudi-based team members (TA and NA), a list of likely organisations/institutes, that may have a role in the future implementation of pharmacy-based services, was compiled. Examples included representatives from the Saudi Pharmaceutical Society and the Saudi Food and Drug Authority (Policy and Planning Division).

Using publicly available contact information, invitations to participate and project information/consent forms were sent via emails addressed to a key officeholder/or their office generally. In this invitation, the key officeholder (e.g., the Manager/CEO/President) to whom or to whose office the invitation was addressed was invited to participate, if they were willing to do so themselves, or to forward the invitation (and attached Participant Information Sheet (PIS)/Consent Form (PCF)) to relevant staff within their organisation/institute. Individuals interested in participation were then able to contact the research team directly using the contact information on the PIS. Both the PIS/PCF clearly outlined that participating institutions or participating members of institutions/organisations would not be identifiable in any way and all interviewee data would be anonymous. An email/phone reminder was sent if there was no response 2 weeks after the initial invitation. In addition, a passive snowballing approach was used by asking consenting participants to recommend other potential interviewees with policy/health service implementation experience to participate in this study. These nominees were then also emailed the project information (PIS and PCF), and the researchers contacted them subsequently to gauge interest in participating.

### Data collection

Semi-structured interviews were used for data collection between December 2018 and February 2019. Interviews were audio-recorded along with note taking and conducted face–face or via phone at a location based on the participant’s preference (e.g., workplace or home) in English by the primary researcher HA (a male Saudi PhD candidate trained in qualitative research) using an interview guide (Additional file 2: Appendix B). Consent to record was obtained before each interview. Sample size for this study was set to be determined by data saturation. In general, in qualitative research, such data saturation requires approximately 20 participants in a relatively homogenous group [23]. All interviews were de-identified and transcribed verbatim before data analysis.

### Interview guide

The interview guide was developed based on our previous findings from other primary/secondary stakeholders [17–19] and our team’s local knowledge (TA, NA), clinical (IK) and health services research experience (BA, BC and IK) and the literature on policymaker perspectives around novel health services implementation. Donabedian’ [24] conceptual framework for evaluating healthcare quality was also used to design prompts within the guide. This model conceptualises three essential fundamentals of a good quality service, which include 1. Structures, 2. Processes, and 3. Outcomes. This guide was pilot tested with three experts before commencing the study to ensure clarity of questions.

### Data analysis

All transcripts were entered in QSR NVivo 11 Software (QSR International, Cambridge, MA) to help with data management and analysis. Independent data coding of two transcripts (10%) was performed by HA and BS, then initial codes were discussed, and an agreed coding framework was developed. Coding was continued with the remaining transcripts by the principal researcher (HA) using this framework. After coding, identified themes and subthemes were discussed with all research team members, with reference to transcript data and a final thematic structure agreed upon by consensus.

## Results

A total of 23 participants (consisted predominantly of males) from both government (Govt) and non-government (NonGovt) policy sectors were interviewed via phone (78%) or in face–face interviews. Of these, almost 65% had pharmacy qualifications and most (16) participants were from the central region (Riyadh, capital city). Ten participants were recruited from academia (Public Universities), nine were from government health sectors and four were recruited from the private sector (i.e., community pharmacy CEOs and private companies). The government employees included representatives from the MoH, the Saudi Food and Drug Authority (SFDA), the King Fahad Medical City (KFMC) and the Ministry of National Guard Health Affairs (NGHA). Data saturation became evident at the completion of interviews with 21 participants, though two more interviews were undertaken to verify this as they had been pre-arranged. Interviews lasted between 24 and 50 min with a median length of 35 min. Participant characteristics are shown in Table 1.

**Table 1:** Participant’s characteristics

Characteristics	Variables	n = 23 (%)
Gender	Male	20
	Female	3
Age bracket (years)	30–39	11
	40–49	6
	50–59	4
	60–69	2
Experience level (years)	≤ 5	7
	6–10	2
	11–15	3
	16–20	4
	> 20	7
Workplace location (city)	Riyadh	16
	Dammam	3
	Al-Qassim	2
	Jeddah	1
	Tabuk	1
Professional Background	Medicine	6^a^
	Pharmacy	15^b^
	Public Health	2
Academic qualifications	Bachelor	7
	Master	6
	Doctor of Philosophy (PhD)	10

^a^4 physicians were specialised in public and community health

^b^One pharmacist had a Master of Public Health

Four themes emerged from the data analysis: (1) Future pharmacy CVD health service models; (2) Need for demonstrable outcomes; (3) Professional engagement and advocacy; and (4) Implementability. Each theme is described below along with illustrative verbatim quotes (and participant’s details, i.e., gender, age bracket, Govt/NonGovt sector).

During interviews, participants referred to both primary and secondary CVD risk prevention services. They also discussed a range of recent public health initiatives implemented by the MoH and SFDA in the context of CVD (tabulated in Additional file 3: Appendix C). This discussion resulted from the interview guide, where the question about current initiatives was placed at the beginning of the interview, as an ice-breaker and to create the setting for the topic of focus.

Similarly, when asked about current pharmacist-supported roles in CVD prevention, participants were aware of and highlighted several roles/services, such as patient education, medication management and monitoring that were currently offered to some extent by clinical pharmacists in some tertiary hospital outpatient clinics. Apart from roles in cardiovascular health, diabetes patient education and blood pressure monitoring were mentioned as services known to be offered in a few community pharmacies (e.g., big chain pharmacies in large metropolitan cities) in Saudi Arabia. While the provision of these pharmacy services has had recent approval from the MoH, participants acknowledged that their current availability is very limited.

## Theme 1: future pharmacy CVD health service models

One of the main aims of the study was to obtain a clear picture about the pharmacy health services model that participants envisaged if they supported the notion of pharmacist provided CVD risk prevention/management services. Of foremost importance is the finding that all participants supported pharmacy preventive health services and indicated that as policymakers they had a general expectation that community pharmacists are capable of running CVD risk screening services.

### CVD health service roles

Specific public health roles that pharmacists could provide described by participants included: (1) health screening and (2) primary (health education on risk factors, identifying individual risk factors), and (3) secondary prevention services (patient education on medications, adherence, lifestyle modifications for those with established CVD).

#### Health screening

While some participants supported the notion of screening using clinical measurement devices to check various CVD parameters (i.e., POCT), others thought simple, fast and uncomplicated screening services, i.e., questionnaire-based risk assessment only, utilising smart technology (applications/apps) in community pharmacy might work better and be more readily accepted by the public.

*“I expect them [community pharmacists] to do just the screening services [e.g. POCT] or provide advice regarding dietary habits, exercise, smoking, and other health promotion and preventive advice. They can discover some cases during the screening. They will have a system connected, to advise patients to go to the nearest PHC [primary healthcare centre] … (Pt1, M, 40–49, Govt.)*

#### Primary prevention

Most participants suggested that pharmacists could adopt active primary prevention screening roles, such as assessing patients’ risk factors, providing targeted CVD education and referrals. A few participants preferred a more minimalist approach, where pharmacists simply provided consumers with information leaflets or brochures, and if needed referred them to a physician or other HCPs for further investigations and education, without conducting risk assessment or patient education activities.

*“I think pharmacists shouldn’t be providing the health promotion advice. I think he is representing a point of contact; so, he is the most accessible person, he does the screening, probably can give the patient a brochure or something and then direct them to somebody who is willing to spend time to give him more advice about the health style, which is usually health educators job.” (Pt15, M, 40–49, Govt.)*

#### Secondary prevention services

Most participants saw merit in pharmacist-provided medication therapy management (MTM) and other medication review services for patients who may already be diagnosed with/prescribed medication for CVD (i.e., secondary prevention) [note: MTM is a service in which a pharmacist conducts a medication use intreview with patients on polypharmacy/long-term medication use to help optimise regimens for safety and efficay [25, 26]]. In fact, some participants suggested that MTM services in patients not yet diagnosed with CVD could be used as an opportunity to undertake CVD risk screening.

*“Medication therapy management (MTM)…. Give people an opportunity to be screened by a point of care machine that gives you the result in a minute for HbA1c, fasting blood glucose or blood pressure…. So, you could screen a lot of people in no time….” (Pt7, M, 30–39, Govt.)*

However, a few participants were reticent to have community pharmacists involved in CVD management aspects.

*“….. In general, they can provide counselling and educate, but management is a red line.” (Pt21, M, 50–59, Govt.)*

### Pragmatic factors

In the operationalisation of such models, however, participants emphasised that professional boundaries needed to be maintained and respected for patient safety.

*“……it seems like the roles of different health professionals in Saudi Arabia are not very clear and the majority of providing health services are focused on physicians, which I think is not the right way currently…” (Pt23, F, 30–39, Govt.)*

Most favoured a collaborative doctor–pharmacist model, where consumers at risk could be referred (perhaps via a connected system with primary healthcare centres) by a pharmacist provider with a written report or risk assessment sheet to either primary care physicians, specialists, another HCP, emergency department/s or even government-maintained hotline support numbers to contact for further help. Pharmacist referral straight on to a specialist was suggested to ease the process of these services, which could be favoured by health consumers. Medical oversight and collaboration around pharmacist provided services were seen as mandatory elements by many participants. Some participants specified the direction that patient care pathways need to take in such collaborative models, as well as the tasks that different professionals should perform. Participants highlighted the need for legislative changes to allow for CVD risk prevention collaborative physician–pharmacist service implementation (e.g., POCT for risk screening and management).

*“If we say how we can improve collaboration, so this can go through enforcement by law. This is number one. Number two is doing workshops and orientation and mixing people and creating a model that people will follow.” (Pt15, M, 40–49, Govt.)*

*“…So, if we said the pharmacist will be responsible for prevention, the next step has to be referring to a specialised physician. If you make a screening system that’s too long for the patient, the patient will hate it. So, if you say primary care physician then specialist, that would be okay. If you say pharmacist then specialised physician, that would be okay. But pharmacist then primary care physician then specialised physician, patient won’t use that model.” (Pt15, M, 40–49, Govt.)*

*“… So, once a patient is seen in the pharmacy and the problem is identified, then the pharmacist needs to send a letter stating what he/she has found and his/her recommendations to the primary care provider. A copy of the pharmacist report shall be given to the patient as well. So, I’m talking here also about medication therapy management side intervention in case of uncontrolled chronic conditions, but that may work for CVD risk assessment.” (Pt2, M, 30–39, Govt.)*

**Fig. 1:**
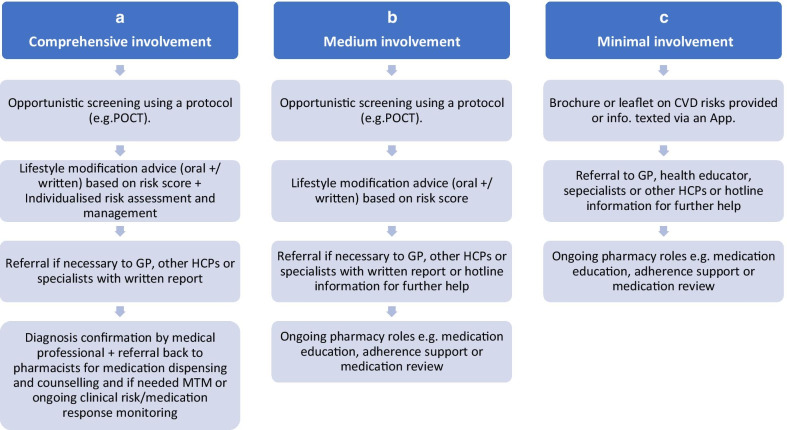
Perceived CVD risk prevention service models of care

The most common future CVD preventive service models in community pharmacy as envisaged by most participants are summarised in Fig. 1. Distilling participant descriptions of preferred service models clearly illustrated three potential levels of pharmacists’ involvement.

Participants recommended that a culturally appropriate screening and risk assessment protocol and supporting tools needed to be developed to maximise service practicality and result in reliable outcomes. These recommendations were made as several participants were aware of assessment and screening tools developed through epidemiological research in other countries (e.g., the Framingham Risk Score [27], which some participants mentioned) that have not been validated in the Arabic language or with Saudi people. Other pragmatic suggestions for pharmacist provided CVD risk prevention/management services related to the physical practice environment. Appropriate service area design (private, comfortable physical space), and access to patient electronic medical records in community pharmacies were two factors recommended to facilitate pharmacist-led interventions.

*“We need to reallocate, adapt, modify, translate, and validate those screening tools in order to make them suitable for our country.” (Pt20, M, 50–59, Govt.)*

*“…if pharmacies can provide such services, there should be a space [a private area in the pharmacy] provided where the pharmacist can sit down with the patient, take the vital parameters… then see what medications the case is taking and sit and discuss management with him or her and to follow up.”(Pt13, M, 60–69, NonGovt.)*

Participants also mentioned the potential benefits that would likely accrue from the recently launched pharmacy initiative, the “Wasfaty Scheme” [a governmental funded initiative that is gradually being rolled out to electronically connect assigned community pharmacies and public primary healthcare centres or hospitals for e-scripts [28]]. It was suggested that the system be utilised for preventive services by supporting assigned community pharmacies to offer CVD risk prevention services.

*“Wasfaty is already applied and available in some pharmacy mainly in the Eastern region of Saudi and across other regions of the country….” (Pt12, M, 30–39, NonGovt.)*

*“In the new model of care, we have different changes that will happen in the system…. So, one of these changes, which we have just started, is that any patient in primary care centres now will get the prescription and he can go to any private pharmacy to take those medications and how about if we advise those assigned pharmacies to do part of the education and screening services…. We should plan to add these screening services as roles for them.” (Pt1, M, 40–49, Govt.)*

## Theme 2: need for demonstrable outcomes

Most participants highlighted that any future community pharmacy CVD risk prevention/management studies needed to be supported by ‘evidence’ before implementation policies could be launched. Sources of such ‘evidence’ mentioned included systematic literature reviews (global literature) and local demonstration projects.

*“… I suggest that the pilot study be done in different socioeconomic levels of the community …. it should be diverse; it should take sample size from different levels of the society.” (Pt6, M, 40–49, Govt.)*

These projects would need to be shown to have concrete evidence of benefit. Benefits sought were demonstrable high-level outcomes, such as risk exposure reduction, lowered mortality and hospital admission rates or cost savings, clinical outcomes and patient reported benefits/satisfaction. A strong health services understanding in our participant group was also evident, as they suggested that well worked out processes/documentation methods to collect data would be required to prove the benefits of pharmacist-delivered preventive services.

*“You can document your success in such initiatives or such intervention by following up with patients who were exposed to such services for years and see what impact that has on their clotting time, on their A1C, on their blood pressure readings. And you can link the reduction to detect a decline in the number of mortalities, of case fatalities or number of hospitalisations in the future….” (Pt10, M, 30–39, Govt.)*

Participants understood that the collection of such evidence would take time and were careful to differentiate between short (e.g., changes in Haemoglobin A1C (HbA1c), BP, cholesterol level) and long-term (reduction in national annual CVD-related hospital admissions and lowered CVD related mortality) benefits expected from such services.

In identifying cost as a key indicator, many participants mentioned the need to use digital health innovations, such as screening applications. They believed that using such technologies could expedite the process for both patient/provider and render it more cost effective. All participants envisioned the long-term savings likely to ensue from these programs and saw expenditure allocation to preventive health as a long-term investment.

*“….. from a commercial view, each $1 spent now in the prevention will save $7 in ten or twenty years in the future for the general cost of the healthcare system and other financial expenses made into healthcare or activation of population….” (Pt1, M, 40–49, Govt.)*

*“Well, if you decrease the number of people who had the complications, then that will cost the health system less in the future, and that will lead to less hiring of people to take care of complications and less expenditure on treatment of these complications.” (Pt15, M, 40–49, Govt.)*

While the participants clearly outlined the need for accumulating evidence supporting pharmacy CVD risk prevention/management services, they were not clear about who would do this research and how it might be funded.

## Theme 3: professional engagement and advocacy

Professional organisations were mentioned as salient foci for the development of pharmacy services, but were described as ‘inactive’ in the Saudi pharmacy profession. Participants recommended that a collection of professional not-for-profit or private organisations should be actively engaged to provide recommendations for pharmacy practice advancement. Collaboration between pharmacy professional bodies and other medical or specialised bodies to develop new patient care models was also seen as essential. Health services models for pharmacy implementation would need stakeholder impetus for sustainability, where the stakeholder body was seen to represent pharmacy educators, private pharmacy business owners, medical professionals, clinical experts and consumers.

*“… I think our professional bodies are still evolving…. What I recommend is an inter-professional model meaning that a professional organization such as the Saudi Pharmacy Association…. They should collaborate with different medical professional bodies such as the Saudi Society of Management of hypertension…and public societies…. advocating for the public. I think this will be more fruitful….” (Pt10, M, 30–39, Govt.)*

*“Well, it requires people in academia, people like us in NGOs, it requires influencers in the community and it requires many sessions of collaboration and workshops to get people who are in the seat of decision making to loosen to the subject matter experts to see the value added in all of the things they want to do.” (Pt13, M, 60–69, NonGovt.)*

A key role that pharmacy professional bodies would need to undertake was to work out legal systems and professional indemnity for pharmacists venturing to the unchartered territory of preventive health service provision.

## Theme 4: implementability

### Professional pharmacy governance

Change in professional governance, workforce development, professional policies and guidelines was an issue stressed by most participants, given that most recognised the limited spectrum of services offered in only few community pharmacies currently.

#### Pharmacy careers and workforce

The need to enhance the profile of community pharmacy careers was highlighted by all participants, given that there was very little engagement in this role by local graduates. It was recognised that higher pay rates, paid leave provisions, job security, professional development could lead to better retention in community pharmacy careers—and that these aspects should be internally regulated by the pharmacy industry. Many participants also posited that boosting professional service roles may be another way of ensuring job satisfaction.

*“We should change the theme/image in Saudi Arabia. This is currently a very unattractive working place, like a supermarket, just selling products…. Also, the salary and incentives for the pharmacists themselves are low. Also, the job security, as community pharmacies are privately-owned and they care about financial profits. They are not willing to give high salary or vacations to pharmacists compared to the governmental sectors. So, there is a need to incentivise those pharmacists to attract them to provide services.” (Pt19, M, 30–39, Govt.)*

Policies suggested by most participants that would be needed to underpin the widened pharmacy service scope included support for Saudization (local instead of expatriate workforce) and gender equity of community pharmacists. Mandating community pharmacy employment for at least 2 year post-graduation for local pharmacy graduates was suggested by a few participants, as it was felt that this may develop future (local) pharmacy graduates’ ongoing engagement with this career role.

*“…We start now with Saudization …. and in my opinion, I see that community pharmacists are mostly expats so if they would be replaced by Saudi male and female pharmacists who will make a change in the way pharmacy is evolving…” (Pt3, M, 60–69, NonGovt.)*

*“It could be done in a way that in the first two years of graduates’ careers they have to work in a community pharmacy after graduation and before going to a secondary or tertiary hospital….” (Pt15, M, 40–49, Govt.)*

#### Pharmacy curricula and ongoing education

Pharmacy curricula content and increased community pharmacy placements were suggested to motivate and equip local pharmacy graduates to work in community pharmacy; this was seen as particularly important for new entrants into the profession.

*“… we might concentrate on fresh graduate students because those fresh graduate students have good knowledge and they are well trained and maybe offering those services will be run by them more than old pharmacists because attitude is difficult to change their practice….” (Pt19, M, 30–39, Govt.)*

Participants also advocated for a better alignment between Saudi pharmacy curricula and pharmacy practice—with skills, such as public health methods, patient psychology, communication and management lacking in the current highly clinical/scientific curricula. As such, it was believed preventive medicine content was under-emphasised in curricula, where the focus remained on pharmacology and therapeutics.

*“The medical/pharmacy education system is very much curative-oriented not preventive-oriented…. the medical or health education system does not focus on the social part or the social determinants of health and how to address those social determinants. These are very poorly addressed in our education….” (Pt20, M, 50–59, Govt.)*

Training for pharmacists already in practice was recommended. It was suggested they would need skills in CVD risk screening and management services, and that such upskilling should be the remit of authorised bodies accredited and committed to providing continuing professional development (CPD). It was thought that such CPD needed to emphasise both clinical and communication skills.

*“…give them enough training before they start the service. And this can be done either through the Saudi Commission, through universities, or through the Ministry of Health. There are several certifications they need to get.….” (Pt11, M, 30–39, Govt.)*

*“…. the training should focus on proper communication. I have to emphasise this many time because proper communication between the healthcare provider or the pharmacist and the patient is a cornerstone in providing this service and for it to work properly.” (Pt4, F, 40–49, Govt.)*

#### Practice policies, standards and guidelines

Participants mentioned that at a policy level, the profession would need to build and support adherence to stringent quality control requirements for pharmacies accredited to provide services. Structurally, a common suggestion was to have a ministerial department appointment for program director that would oversee pharmacy delivered CVD risk prevention services. A few participants mentioned third party inspection and quality control certification bodies. A current lack of quality accreditation bodies/systems for community pharmacy practice was also seen as an issue that needed to be addressed; for example, through CBAHI (The Saudi Central Board for Accreditation of Healthcare Institutes) Surveyors.

*“… the role of healthcare leader is to (a) standardise their work, (b) monitor the quality or output of their work, and (c) evaluate and compare between different sources of information or those of pharmacies with other members of healthcare team.” (Pt1, M, 40–49, Govt.)*

*“…… The Ministry of Health can have a contract with a third party who is going to assess the quality of services delivered by the pharmacies and then that would be connected with a reimbursement given to such services.” (Pt16, M, 30–39, Govt.)*

*“Not all of them [community pharmacies] are ready because up to now there are no standards of accreditation for community pharmacy. If they put accreditation standards and include it, most of them will be ready.” (Pt8, M, 50–59, Govt.)*

### Implementation vision

#### Scalability

Participants suggested that health services models constructed would need to be scalable. A popular idea was that utilising the bigger pharmacy chains as initial ‘test’ sites would allow a quick scale up, should the models have demonstrable positive outcomes and acceptability. It was also recognised that implementation may need to be incremental rather than nationwide rollout at one go. It was suggested that the initial implementation could take place in chain pharmacies, as they are more ‘ready’ to provide such services, while wider national implementation needs time.

*“…We would start with big chain pharmacies to provide those services like for example in Riyadh we have like 2 or 3 pharmacies that might want to start those services and start gradually and this eventually will end up with completing those services in all of those pharmacies ….” (Pt19, M, 30–39, Govt.)*

*“… I suggest that this be at least mandatory for chain pharmacies. For example, now XX chain pharmacy have almost 1000 pharmacy, we can make it mandatory that of every 50 pharmacies, at least one should provide screening. If they wish to increase this limit, it would be up to them, but this shall be the minimum.” (Pt8, M, 50–59, Govt.)*

#### Affordability and access

Participants suggested health promotion/social marketing methods to enhance the public’s awareness of these services, using a range of marketing channels (social media, referrals (health professionals), word of mouth, television and radio). It was thought that such measures were especially important to create a reach for pharmacy services in rural areas—since access to physicians in such areas is limited. Public health activists, patients’ advocates, social media influencers and religious leaders were also suggested to be engaged to enhance awareness of these services.

*“By educating the public through social media like WhatsApp…. and through television and radio….” (Pt14, M, 30–39, Govt.)*

*“….in rural areas, where there is a lack in number of physicians, pharmacists are well-trained and educated to provide the necessary preventative measures….” (Pt2, M, 30–39, Govt.)*

*“…. men of religion or leaders can help address the issue [CVD risk]. They can talk to people in mosques about the Islamic way of living as a good system or a good model to prevent those problems because our religion, Islam, is always in favour of prevention of CHD [Coronary Heart Disease]. This has to be utilised.” (Pt20, M, 50–59, Govt.)*

Participants emphasised that incentives for service-provider pharmacists were required for the sustainability of such services. A reimbursement system via the government and/or health insurance companies was suggested (on a pay-for-performance basis). This, it was proposed, would keep the services affordable for patients.

*“…. So, if you want to implement screening, then it has to be paid for either by the government or the insurance companies….” (Pt15, M, 40–49, Govt.)*

*“…. Cost affordability of the services will encourage patients to come and use these services….” (Pt15, M, 40–49, Govt.)*

#### Service adoption

The participants mentioned that as public perception of community pharmacists was still centred around them being ‘products suppliers’, there may be hesitancy in engaging with pharmacy services. High-quality, standardised, ministry supported/accredited services were likely to, over time, address consumer hesitancy in engaging with pharmacy services by accruing trust.

*“…the most important aspects are quality standards that have to be met in screening to establish a very strong and trustworthy patient-pharmacist relationship and this will be established by more communication between the pharmacist and patients….” (Pt4, F, 40–49, Govt.)*

*“… many people look at pharmacies as a place where they go, buy things, and go out. They don’t think of pharmacies as healthcare facilities. This, I think, will be one of the limitations that will exist, initially at least. They need to readjust their way of thinking that pharmacists are just vendors….” (Pt2, M, 30–39, Govt.)*

#### Health system changes

Most participants agreed that the Saudi primary healthcare system was still evolving. The system lacked current networking paths between community pharmacy and primary healthcare centres; these would need to be facilitated to implement pharmacy CVD risk prevention/management. Some physicians were thought to still be unaware of pharmacists’ role and ability in general and particularly community pharmacists, which may impact policy and regulations for pharmacy practice.

*“Well, I think the primary healthcare system in Saudi Arabia is still fragmented….” (Pt10, M, 30–39, Govt.)*

*“……Currently, there is a complete lack of understanding and lack of knowledge among physicians and other healthcare professionals, to be honest, about the role of pharmacists in clinical and community settings….” (Pt2, M, 30–39, Govt.)*

**Fig. 2:**
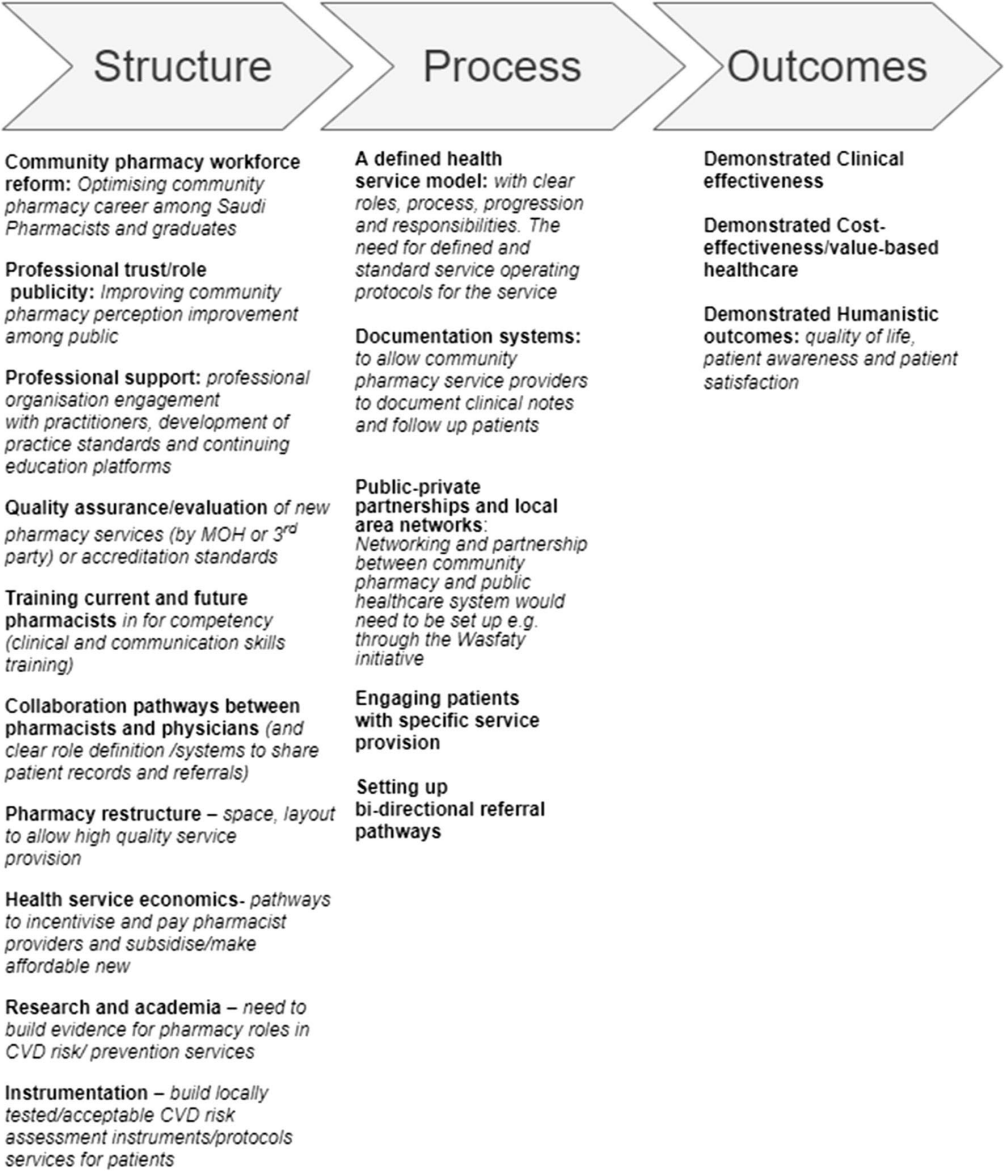
Potential Saudi pharmacy CVD risk prevention/management service—service elements that would need to be in place prior to implementation

Finally, as the interview guide applied Donabedian’s model for service quality [24], which identifies three key elements of a high-quality health service (structures, processes and outcomes), the raw data were examined to see the extent to which participant discussions centred around these factors (Fig. 2).

## Discussion

This is the first study to elicit policymakers’ views on the potential for implementation of pharmacy CVD risk prevention/management services in Saudi Arabia. It was clear that while policymakers/opinion leaders supported the initiative in principle, significant work on developing the structural elements for such services was perceived to be needed. These structural elements would require deep-rooted reforms at many levels—legislative, social, professional and fiscal. Policymakers indicated the need for demonstrable outcomes for such pharmacy health services prior to supporting widespread implementation. These findings concur with policymakers globally [29, 30]. It is also apparent that much work is expected from academia (research and pharmacist training) and pharmacy professional bodies before pharmacy CVD risk prevention/management services can be proposed for implementation at ministerial/policymaker levels. The research findings provide a blueprint for Saudi pharmacy health services researchers to progress upon in the coming years.

Pharmacy provided CVD risk prevention/management services were generally supported by participants, and models envisioned for such services ranged from minimal to comprehensive pharmacist involvement. Such variation in perspectives about the nature and complexity of pharmacy health service models have been observed in other studies [31].

In interpreting and translating variable stakeholder concepts, pharmacy health service researchers often test various versions of a novel service. For example, two different models of pharmacy involvement (e.g., basic vs. comprehensive) in CVD risk, diabetes and sleep disorders screening were developed and tested by Australian pharmacy health services researchers [32–34].This approach might be adopted in the Saudi context, for example, by testing different pharmacist provided CVD risk prevention and management service models in parallel, using robust research designs, with outcomes, such as screening efficiency/diagnostic accuracy, cost effectiveness, as well as implementation potential. From a scalability standpoint, another approach may be to develop and test a CVD risk prevention/management model with minimal pharmacist involvement and implement this initially. Over time, with the development of pharmacists’ skills and interprofessional collaborative experience, broader acceptance by both pharmacists and their health professional colleagues for an increased pharmacist involvement in service models with greater clinical decision making, might be achieved. Initial testing and implementation of a novel pharmacist risk prevention/management model could be trialled in larger chain pharmacies which offer a large patient/consumer base for testing model efficacy, and structures and process controls enabling them to embed novel health services into routine pharmacy practice leading to subsequent scale-up. A key enabler to service implementation should be the development of professional practice standards by the profession in Saudi Arabia, including a specific set of CVD risk prevention/management practice standards. This would facilitate national wide uptake of health services for chronic diseases at a foundational level and serve as a platform for more complex health service models in the future.

Policymakers/opinion leaders, who participated in this study, also emphasised that for the government to support implementation of pharmacy CVD/risk prevention services, there would need to be evidence of their benefit to all parties, patients, providers and service funders (insurance companies or government), i.e., clinical, humanistic and economic outcomes [35]. Poor evaluation of community pharmacy services internally (at an organisational level) and limited evaluation at a whole-profession level are known to beleaguer pharmacy service implementation [36, 37]. To enable data collection of service provision and patient outcomes, quality documentation systems would need to be established, given that clinical documentation is not a current strength in the Saudi community pharmacy sector. For example, electronic dispensing programs, though not routinely used in Saudi community pharmacy practice, could be used to record medication supply as well as document interventions and patient outcomes in the context of novel service models. They may also serve as a platform for clinical decision making support, helping to standardise service provision broadly [35]. Given digital health innovations worldwide, e-platforms supporting conventional and novel pharmacy practice in Saudi Arabia are needed. Saudi consumers too, appear to engage with emerging e-health pathways, e.g., use of electronic health records which have been implemented in some public hospitals in alignment with the Vision 2030 [38]. Telehealth and digital technology use by community pharmacists has the potential to improve health outcomes [39–41]. Such initiatives may increase access to screening services and facilitate efficient care of cardiovascular conditions as highlighted in a recent review which describes pharmacist involvement in novel smartphone and web-based screening studies for a range of chronic conditions [40]. Other aspects of virtualisation may include using computerised decision support systems especially by integrating these with electronic dispensing platforms and using the program to document interventions or generate referral letters [42].

Notably these key stakeholders valued and proposed clear pathways to develop a local community pharmacy workforce (given that much of this workforce comprises expatriates currently [43, 44]). Their suggestions aligned with psychological theories of job satisfaction, such as Maslow’s hierarchy of needs [45], where basic needs (pay, leave) must be worked upon and progressed along a clear trajectory of prestige, job satisfaction and professionals’ self-actualisation. These suggestions also align well with feedback from potential incumbents into community pharmacy careers, e.g., late stage/final year pre-registration Saudi pharmacy students, who reported in a national survey that improving salaries and financial income, role prestige and career progression paths in community pharmacy jobs would be factors in considering a career in community pharmacy working in community pharmacy [46]. One way of attracting and retaining local graduates in community pharmacy careers would be to develop health service roles for community pharmacists. Such roles, which require clinical decision making and interprofessional collaboration, were highly likely to lead to a well satisfied community pharmacy workforce. More authoritarian approaches around mandated work for a period of time post-graduation in community pharmacy were also suggested by some participants. These compulsory service arrangements have been used globally by governments to distribute government supported investment in education across underserved areas [47]; though not favoured by many, they have been shown to fulfil workforce distribution goals [47, 48]. Policymakers in Saudi Arabia will need to think carefully about underpinning compulsory community pharmacy work with optimal educational/financial/registration incentives, and to collect evidence whether the ‘community pharmacy exposure’ does indeed eventuate in increased uptake of community pharmacy as a career option by future graduates.

The stakeholders in this study, as did consumers, pharmacists and physicians in previous studies in Saudi Arabia [17–19], recognised the need to build a better image of community pharmacy to enhance its profile for engagement by consumers with any pharmacist provided health services. Indeed, during the Coronavirus disease 2019 (COVID-19) pandemic globally, community pharmacy has served an essential service, in managing medicine shortages, extendable supply based on prior histories in chronic conditions and upscaled handling of minor ailments in areas, where primary care physician facilities were not operating [49–54]. In Saudi Arabia, pharmacists’ skills have been fully utilised during the current pandemic to enhance the safety response [55]. This current wave of public esteem could and should serve as a step towards constructing a positive, trustworthy image of community pharmacy professionals with skills far surpassing mere medication supply. There are scant reports in the literature about profession/government driven publicity drives that serve to inspire trust and engage consumers with community pharmacies as health service provision venues. Such social marketing campaigns for community pharmacy are much needed and timely for advancement of the profession for public health benefits.

Another required structural reform related to pharmacy undergraduate curricula/post qualification continuing education. Balancing a curriculum currently heavily skewed towards pharmaceutical and clinical sciences by incorporating more training around psycho-social aspects of health beliefs and medication use, effective communication and empathetic responsiveness would better fit the health services models explored in this study. This requires national level discussions between key pharmacy education providers and laying down of clear Saudi-based accreditation standards that all providers could follow or base their pharmacy pre-registration programs on. Reported findings from pharmacy educationists align with the recommendations by our study participants [56]. While mentioned in our study in the context of novel pharmacy services, increasing consumer awareness and advocacy will necessitate the inclusion of these soft-skill aspects of health education. Interprofessional education may also promote pharmacists’ skills to other professionals and pave the way for collaborative patient care. Such educational development should be framed carefully around existing curricular gaps and training needs. Although no such scholarly inquiry works in pharmacy education are reported from Saudi Arabia, in a neighbouring country, Qatar, an interprofessional education project focussing on assessment and screening for CVD was offered jointly to a range of health students from medicine, nursing and pharmacy colleges [57]. In this Qatari study, a needs analysis phase comprising student knowledge surveys was undertaken, where it was clear that curricular lacunae existed in terms of patient assessment skills in participating students [57, 58]; this consequently led to an inter-disciplinary curriculum review. Qatari researchers also specifically demonstrated competency gaps in a sample of community pharmacists around CVD risk assessment and management using a simulated patient study [59], and developed a pedagogically sound targeted education program for pharmacists to enhance their competence in providing CVD risk assessment and management [60].

**Fig. 3:**
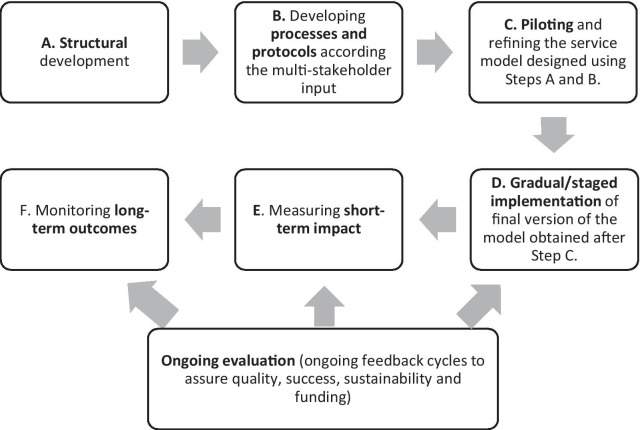
Implementation steps suggested

As mentioned in the introduction, perspectives from multiple stakeholder groups have now been explored by our research group (i.e., pharmacists [18], health consumers [17], physicians [19]), and adding to them the findings of this study (i.e., policymakers viewpoints), we propose a possible schema for implementing high-quality CVD risk screening and management services in community pharmacy as shown in Fig. 3. A detailed implementation plan utilising an implementation science framework is recommended to be developed and discussed with or sent to key stakeholders, e.g., from MoH and private sector, and consider any feedback.

The strength of this study is that it lays the foundational recommendations of policymakers/opinion leaders with regards to implementing high-quality health services in Saudi community pharmacy. Although most participants in this study were from the central region (Riyadh), the sampling techniques targeted and achieved a range of age groups, experience, workplace and sector representation (government and private) to ensure variability and representativeness [61]. Quality criteria for qualitative research were followed, although we could not undertake member-checking (participants verifying transcript as true record of interview) for logistic reasons; however, interview transcripts and records were reviewed for accuracy by the primary researcher. Nonetheless, this may lead have led to a biased interpretation of participants intent.

## Conclusion

To implement high-quality CVD risk screening and management services in Saudi community pharmacy requires a systematic approach, applying the principles of implementation science. Three possible CVD risk screening and management service models were identified: minimal pharmacists’ intervention, medium level intervention, and comprehensive intervention. It was recommended to mandate the minimal service model as a practice standard for Saudi community pharmacists and over time evolve to the other two service models.

Findings revealed more structural reform needs compared to process development or outcome measurement planning at pharmacists/pharmacy, patient and health-system levels, is not surprising, as the Saudi primary health system is still evolving. Health-system reform in terms of facilities, resources and more involvement of community pharmacy within public primary health system are necessary. Pharmacy practice curricula reform will be essential to address the mismatch between academia and practice and to encourage and prepare pharmacy graduates to consider community pharmacy as a career path. Professional support will be vital in providing continuous training, advocating for the profession of pharmacy and building collaboration with other organisations to expand pharmacists’ roles and opportunities. Prior to implementation, all enablers/barriers identified by stakeholders must be addressed to ensure quality and ultimately sustainability of patient-centred services provided in community pharmacy.

## Supplementary Information

**Additional file 1.** Consolidated Criteria for Reporting Qualitative Studies (COREQ) checklist.

**Additional file 2.** Interview Guide.

**Additional file 3.** Recent policy initiatives by Saudi MoH and SFDA within the last 3–4 years.

## Data Availability

The data collection guide and COREQ check list used during this study are attached as additional files in Additional file 1: Appendix A and Additional file 2: Appendix B.

## Abbreviations

BP: Blood pressure
CBAHI: The Saudi Central Board for Accreditation of Healthcare Institutes
CHD: Coronary Heart Disease
COREQ: Consolidated Criteria for Reporting Qualitative Studies
COVID-19: Coronavirus disease 2019
CVD: Cardiovascular disease
Govt: Government
GP: General practitioner
HCPs: Healthcare professionals
HbA1c: Haemoglobin A1C
HREC: Human Research Ethics Committee
IRB: Institutional Review Board
KFMC: King Fahad Medical City
MoH: Ministry of Health
MTM: Medication therapy management
NDCs: Non-communicable diseases
NGHA: Ministry of National Guard Health Affairs
NonGovt: Non-government
PCF: Participant consent form
PIS: Participant information sheet
POCT: Point of care testing
SFDA: Saudi Food and Drug Authority
