# Supplementary material for: Cardiovascular disease risk prevention services by pharmacists in Saudi Arabia: what do policymakers and opinion leaders think?
Source: J Pharm Policy Pract. 2021 May 6;14:42. doi: 10.1186/s40545-021-00319-6 (PMC8100751; doi:10.1186/s40545-021-00319-6)
Supplement: Supplementary file 2 — Additional file 2. Interview Guide. [file 40545_2021_319_MOESM2_ESM.docx]

Interview Guide

-Policymakers/Industry Leaders/ Health Services Implementors

**Interviewee ID………….. (Research provided ID number)**

**Date …../……/……….,**

**Section A: Pre-interview procedure**

The interview will begin by highlighting to the participant(s) demonstration examples of studies from various global contexts where pharmacists have provided CVD risk prevention and management services. This demonstration material will be in an infographic presentation style and either Power Point slides or a printed small booklet will be the format used to showcase these examples, so that participants gauge the context of the interviews. This demonstration phase will be followed by a brief summary of key results obtained through the protocol that was approved with patients and providers.

**Section A: Interview Questions**

| **OBJECTIVE / REASON OF ASKING THE QUESTION** | **INTERVIEW QUESTIONS** | **PROBES FOR KEY QUESTIONS (TO BE ASKED ONLY IF NEEDED)** |
| --- | --- | --- |
| **To understand the participants awareness and perspective of what is currently happening** | **1a. What do you believe are the key CVD risk prevention services available in Kingdom of Saudi Arabia (KSA)?** |  |
|  | **1b. In recommending or supporting CVD risk prevention services/programs within KSA, what attributes (quality indicators) would be expected to be met by your department/staff/ ministerial head?** | **Probe about:**   - Cost effectiveness - Clinical effectiveness/efficacy evidence - Acceptability by patient and providers - Delivering health workforce readiness - Key Performance Indicators (probe) |
| **Future feasibility of primary care models** | **2a. Given your position, are you aware of any CVD risk prevention services/programs for the public in KSA that are in planning stages or likely to be implemented in the near future?**   - **Follow up question:**   **2b. What is your opinion about involving pharmacists (community and hospital) in delivery of CVD risk prevention/management services for the public?** |  |
| **Potential health service models** | **3a. There is a view among certain stakeholders in KSA that pharmacists have the potential to provide CVD risk prevention and management services.**  **How do you envisage such models working?** | **Probe about:**   - **Service type**   *CVD risk screening and referral, collaborative models with physicians, point of care testing for CVD risk markers*   - **Clinical oversight/ interprofessional collaboration** - **Legislative and professional support** - **Incentivisation** and **sustainability** issues - Public expectation of pharmacies and **promoting pharmacy based services** - potential **limitations** in such models |
|  | **3b. In contrast, there may be stakeholders who believe that pharmacists do not have the potential to provide CVD risk prevention and management services in KSA. What would your comments be in this case?** | **Probe about:**   - Views about **pharmacy capacity/workforce status** - Public and other profession **role expectations** - **Policies and legislation** - **Health system/ health economic issues** - **Potential advantages of pharmacy models** |
| **Forward planning for pharmacy service models** | **What in your opinion, are the materials/evidence that would be needed to make a strong case for pharmacy to deliver such services?**  **i.e. were we as researchers planning to conduct research to test for evidence, what outcomes should we measure?** | **Probe about:**   - Evidence of **acceptability from stakeholder**s - **Pilot data** on feasibility and effectiveness - **Guidelines** for service provision - Review of **evidence from other countries** - Outcomes of planning meetings and discussions with key stakeholders |
|  | **Following the notion of a hypothetical proposal for pharmacy-based CVD risk prevention and management services, what would you expect of the pharmacy profession to enable this to be implemented?** | **Probe professional development around:**   - **Practice/service provision standards** - Protocols and guidelines for CVD roles - **Accreditation and training** standards - **Audit and quality control** processes and indicators |
| **Stakeholder investment** | **Again, following the notion of a hypothetical proposal for pharmacy based CVD risk prevention and management services, who would be key stakeholders that your department/organisation/staff would be consulting for proposal development?** | **Probe key stakeholders** |

**Section C: About yourself**

**1. Age.............. Years OR age range 🞏20s, 🞏30s, 🞏40s, 🞏50s, 🞏60s, 🞏70s, 🞏 ≥80 years,**

**2. Gender** 🞏Male, 🞏Female **[Interviewer to Note].**

**3. Work City/Region …………**

**4. Job type (Government/ Non-Government)**

**5. Work experience in your current role……… years**

**6. What is your background qualification?**

- *Clinical/ public health/ management/ law/ other (Please specify) ………*

**7. What is the highest degree you have attained?**

- *Diploma*
- *Bachelor*
- *Master*
- *PhD*
- *Dr Pharm or Pharm D*
- *Other (Please specify) ………*
