# Supplementary material for: Cardiovascular disease risk prevention services by pharmacists in Saudi Arabia: what do policymakers and opinion leaders think?
Source: J Pharm Policy Pract. 2021 May 6;14:42. doi: 10.1186/s40545-021-00319-6 (PMC8100751; doi:10.1186/s40545-021-00319-6)
Supplement: Supplementary file 3 — Additional file 3. Recent policy initiatives by Saudi MoH and SFDA within the last 3–4 years. [file 40545_2021_319_MOESM3_ESM.docx]

**Appendix C**

Table1. Recent policy initiatives by Saudi MoH and SFDA within the last 3-4 years.

| **Policy types** | **Public health initiatives** |
| --- | --- |
| **Food labelling and product standards** | Product specifications, labelling standards and calories labelling in restaurants and food providers such as calorific content, levels of Salt (NaCl) and trans-fat etc |
| **Health Education Campaigns** | Awareness and education campaign around dietary recommendations on salt or trans fats reduction |
| **Urban design and planning policies** | Exercise facilities and walking paths and social marketing via social media |
| **Fiscal measures** | Taxation on tobacco products |
| **Healthcare programs – tertiary sector** | Allowing some clinics to provide health awareness programs |
